# Deep Attention Models for Human Tracking Using RGBD

**DOI:** 10.3390/s19040750

**Published:** 2019-02-13

**Authors:** Maryamsadat Rasoulidanesh, Srishti Yadav, Sachini Herath, Yasaman Vaghei, Shahram Payandeh

**Affiliations:** 1 Networked Robotics and Sensing Laboratory, School of Engineering Science, Simon Fraser University, Burnaby, BC V5A 1S6, Canada; payandeh@sfu.ca; 2 School of Computing Science, Simon Fraser University, Burnaby, BC V5A 1S6, Canada; sachini_herath@sfu.ca; 3 School of Mechatronic Systems Engineering, Simon Fraser University, Burnaby, BC V5A 1S6, Canada; yvaghei@sfu.ca

**Keywords:** computer vision, visual tracking, attention model, RGBD, Kinect, deep network, convolutional neural network, Long Short-Term Memory

## Abstract

Visual tracking performance has long been limited by the lack of better appearance models. These models fail either where they tend to change rapidly, like in motion-based tracking, or where accurate information of the object may not be available, like in color camouflage (where background and foreground colors are similar). This paper proposes a robust, adaptive appearance model which works accurately in situations of color camouflage, even in the presence of complex natural objects. The proposed model includes depth as an additional feature in a hierarchical modular neural framework for online object tracking. The model adapts to the confusing appearance by identifying the stable property of depth between the target and the surrounding object(s). The depth complements the existing RGB features in scenarios when RGB features fail to adapt, hence becoming unstable over a long duration of time. The parameters of the model are learned efficiently in the Deep network, which consists of three modules: (1) The spatial attention layer, which discards the majority of the background by selecting a region containing the object of interest; (2) the appearance attention layer, which extracts appearance and spatial information about the tracked object; and (3) the state estimation layer, which enables the framework to predict future object appearance and location. Three different models were trained and tested to analyze the effect of depth along with RGB information. Also, a model is proposed to utilize only depth as a standalone input for tracking purposes. The proposed models were also evaluated in real-time using KinectV2 and showed very promising results. The results of our proposed network structures and their comparison with the state-of-the-art RGB tracking model demonstrate that adding depth significantly improves the accuracy of tracking in a more challenging environment (i.e., cluttered and camouflaged environments). Furthermore, the results of depth-based models showed that depth data can provide enough information for accurate tracking, even without RGB information.

## 1. Introduction

Despite recent progress in computer vision with the introduction of deep learning, tracking in a cluttered environment remains a challenging task due to various situations such as illumination changes, color camouflage, and the presence of other distractions in the scene.

One of the recent trends in deep learning, attention models, are inspired by the visual perception and cognition system in humans [1], which helps to reduce the effect of distraction in the scene to improve tracking accuracy. The human eye has an innate ability to interpret complex scenes with remarkable accuracy in real time. However, it tends to process only a subset of the entire sensory information available to it. This reduces the eyes work to analyze complex visual scenarios. This ability of the eye comes from being able to spatially circumscribe a region of interest [2]. By making a selective decision about the object of interest, fewer pixels need to be processed and the uninvolved pixels are ignored, which leads to lower complexity and higher tracking accuracy. As a result, this mechanism appears to be key in handling clutter, distractions, and occlusions in target tracking. 

Illumination changes and color camouflage are two other challenges for tracking algorithms. The emergence of depth sensors opened new windows to solve these challenges due to their stability in illumination changes and not being sensitive to the presence of shadow and a similar color profile. These features make depth information a suitable option to complement RGB information. 

In this paper, a RGBD based tracking algorithm has been introduced. In the proposed methods, a convolutional neural network (CNN) has been utilized to extract two types of feature: Spatial attention features, which select the part of the scene where the target is presented; and appearance features—local features related to the target being tracked. We take advantage of the attention model to select the region of interest (ROI) and extract features. Different models are proposed to select the ROI based on both depth and RGB. In addition, a model is implemented using only depth as the resource for tracking, which takes advantage of the same structure. Our work extended the existing work proposed by Kosiorek et al. [3], which consisted of an RGB-based model that was modified to supplement depth data in four different ways. We evaluated the efficiency of our trackers using the RGBD datasets of Princeton [4], and our own data, collected using Kinect V2.0. During the test, we paid special attention to investigating challenging scenarios, where previous attentive trackers have failed. The results showed that adding depth can improve the algorithm in two ways: First, it improves the feature extraction component, resulting in a more efficient approach in extracting appearance and spatial features; second, it demonstrates a better performance in various challenging scenarios, such as camouflaged environments. We also showed that using depth could be more beneficial for tracking purposes. 

The remainder of the paper is divided into four sections. In Section 2, we examine recent progress and state-of-the-art methods. Methodology and details of our proposed method are explained in Section 3. The experimental setup and results are reported in Section 4. Finally, the paper is concluded and discussed in Section 5. 

## 2. Related Works

Establishing a tracking algorithm using RGB video streams has been the subject of much research. A tracking algorithm usually consists of two different components: A local component, which consists of the features extracted from the target being tracked; and global features, which determine the probability of the target’s location. 

Using a convolutional neural network (CNN) to extract the features of the target being tracked is a very common and effective method. This approach mainly focuses on object detection and the local features of the target to be employed for tracking purposes [5,6,7]. Qi et al. [5] utilized two different CNN structures jointly to distinguish the target from other distractors in the scene. Wang et al. [6] designed a structure which consisted of two distinct parts: A shared part common for all training videos, and a multi-domain part which classified different videos in the training set. The former part extracted the common features to be employed for tracking purposes. Fu et al. [7] designed a CNN based discriminative filter to extract local features. 

Recurrent neural networks (RNN) are also well studied to provide the spatial temporal features to estimate the location of the target in the scene (focusing on the second component of tracking). For example, Yang et al. [8] fed input images directly to a RNN module to find the features of the target. Also, the multiplication layer was replaced by a fully convolutional layer. Long short-term memory (LSTM) was modified by Kim et al. [9] to extract both local features and spatial temporal features. They also proposed an augmentation technique to enhance input data for training.

**Figure 1:**
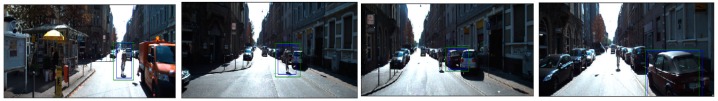
The hierarchical attentive recurrent tracking (HART) [3] algorithm failed to track the cyclist when the color of the background was similar to the foreground in the KITTI dataset [14].

Visual attention method recently gained popularity and are being utilized to extract the local and global features in a tracking task [10,11,12,13]. Donoser et al. [10] proposed a novel method in which they employed the local maximally stable extremal region (MSER), which integrated backward tracking. Parameswaran et al. [11] performed spatial configuration of regions by optimizing the parameters of a set of regions for a given class of objects. However, this optimization needs to be done off-line. An advanced hierarchical structure was proposed by Kosiorek et al. [3], named hierarchical attentive recurrent tracking (HART), for single object tracking where attention models are used. The input of their structure is RGB frames where the appearance and spatial features are extracted. Although their proposed structure is able to track the object of interest in a sequence of images in the KITTI dataset [14] and KTH datasets [15] using RGB inputs, their proposed algorithm failed in more challenging environments (e.g., when there was a similar background and foreground color). Figure 1 shows one of the scenarios in the KITTI dataset [14] which their structure failed to address. We were able to improve the accuracy of their algorithm and overcome the challenges associated with this problem significantly by adding depth. 

There are shown in many situations where only RGB information fails to address accurate tracking in the state-of-the-art algorithms [16,17]. One way to overcome RGB flaws is to complement it with depth for improved accuracy and robustness. The emergence of depth sensors, such as Microsoft Kinect, has gained a lot of popularity both because of the increased affordability and their possible uses. These depth sensors make depth acquisition very reliable and easy, and provide valuable additional information to significantly improve computer vision tasks in different fields such as object detection [18], pose detection [19,20,21] and tracking (by handling occlusion, illumination changes, color changes, and appearance changes). Luber et al. [22] showed the advantage of using depth, especially in scenarios of target appearance changes, such as rotation, deformation, color camouflage and occlusion. Extracted features from depth provide valuable information which complements the existing models in visual tracking. On the other hand, in RGB trackers, the deformations lead to various false positives and it becomes difficult to overcome this issue, since the model continues getting updated erroneously. With a reliable detection mechanism in place, this model will accurately detect the target and will be updated correctly, making the system robust. A survey of the RGBD tracking algorithm was published by Camplani et al. [23], in which different approaches of using depth to complement the RGB information were compared. They classified the use of depth into three groups: Region of interest (ROI) selection, where depth is used to find the region of interest [24,25]; human detection, where depth data were utilized to detect a human body [26]; and finally, RGBD matching, where RGB and depth information were both employed to extract tracking features [27,28]. Li et al. [27] employed LSTM, where the input of LSTM was the concatenation of depth and the RGB frame. Gao et al. [28] used the graph representation for tracking purposes, where targets were represented as nodes of the graph and extracted features of RGBD were represented as the edges of the graph. They utilized a heuristic switched labeling algorithm to label each target to be tracked in an occluded environment. Depth data provide easier background subtraction, leading to more accurate tracking. In addition, discontinuities in depth data are robust to variation in light conditions and shadows, which improves the performance of tracking task compared to RGB trackers. Doliotis et. al. [29] employed this feature of Kinect to propose a novel combination of depth video motion analysis and scene distance information. Also, depth can provide the real location of the target in the scene. Nanda et al. [30] took advantage of this feature of depth to propose a hand localization and tracking method.Their proposed method not only returned x, y coordinates of the target, but also provided an estimation of the target’s depth. They used this feature to handle partial and complete occlusions with the least amount of human intervention.

However, adding depth introduces some additional challenges, such as missing points, noise, and outliers. For instance, in the approach explained by Nguyen et al. [31], first the depth data is denoised through a learning phase and the background is detected based on the Gaussian mixture model (GMM). Following this, the foreground segmentation is accomplished by combining the data extracted from both depth and RGB. In another approach, authors use a Gaussian filter to smooth the depth frame and then create a cost map. In the end, they use a threshold to update the background. Changing color space is also a common approach used to increase the stability of the system. For example, in one approach, color channels are first transferred to chromaticity space to avoid the effect of shadow and then kernel density estimation (KDE) was applied on color channels and the depth channel [32]. Unlike the color channels, which updated background frequently, no background update was necessary on the depth channel due to its stability against illumination changes. In their method, they considered the missing depth points and estimated the probability of each missing point to be part of the foreground. Depth data were used to provide extra information to compensate for the artifacts of background estimation methods based on RGB information. Differences in the depth value of two frames have been utilized to detect the ghost effect in the RGB background subtraction algorithm (i.e., when a moving object is detected in the scene while there is no corresponding real object). Changing color space and transforming three RGB channels to two channels was a method utilized by Zhou et al. [33]. This method gave them flexibility to add depth information as the third channel. Consequently, they applied the new three channels to feed to a common RGB background subtraction method.

Though there have been multiple attempts in using RGB and depth together, using the robustness of depth data by itself has also shown tremendous results. Even in the absence of RGB, depth features are still very distinguishable, making them an important feature which can complement existing visual tracking models. Additionally, using depth by itself has a lot of merits for cases where privacy is important and when one needs to reconstruct the shape and posture of people in the environment. For many applications, providing a tracking method based on only depth can protect the privacy of the target and hence this can be more desirable. Several studies have focused on using only depth data to analyze its strength: Chen et al. [34] employed only depth data for hand tracking; Izadi et al. [35] proposed ‘KinectFusion’, which utilized full depth maps acquired from the Kinect sensors for scene reconstruction; and recently Sridhar et al. [36] presented a fast and reliable hand-tracking system using a single depth camera. In all these cases, the system avoided any reliance on the RGB data, thereby proving that depth by itself can produce robust tracking benchmarks. Haque [37] presented a novel method of person reidentification and identification using attention models, from depth images. 

In this paper, we modified the HART structure proposed by Kosiorek et al. [3] to develop three different methods which included depth data. In the first, three channels of RGB were reduced to two by changing chromaticity space, and depth was fed to the structure as the third channel. For the second, we improved our first model by adding one convolution layer, combining depth and RGB inputs and feeding that to the rest of the network. In the third method, two distinct layers were used to extract the attention features for each of the depth and RGB inputs to select the most dominant features as a feedforward component. Finally, we proposed a CNN to extract both spatial and appearance features by feeding only a depth map to the structure. We evaluated the efficiency of our proposed trackers using the Princeton dataset [4] and our data, collected using Kinect V2.0. During our tests, we focused the investigation on challenging scenarios where previous recurrent attentive trackers have failed. 

## 3. Methodology

**Figure 2:**
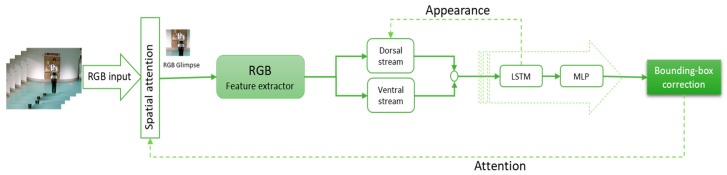
RGB tracker model in [3]. The glimpse is selected in spatial attention module; then, using a convolutional neural network (CNN), the appearance feature spatial feature is extracted and fed to long short-term memory (LSTM).

**Figure 3:**
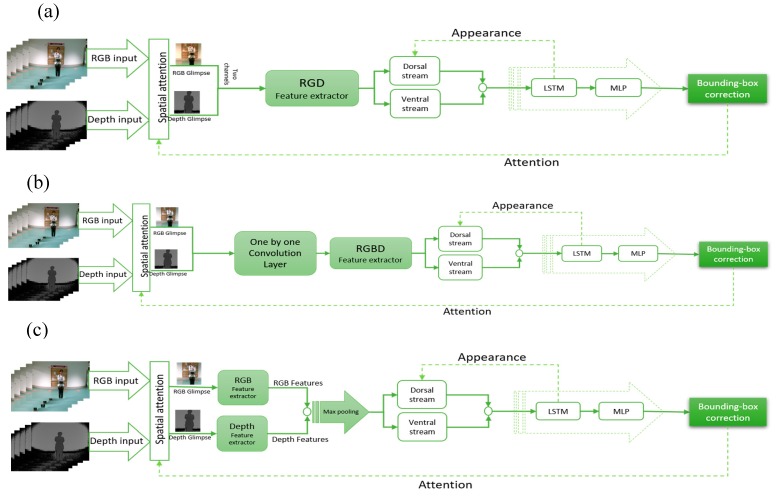
(**a**) RGD Tracker model, (**b**) RGBD combined tracker model, and (**c**) RGBD parallel tracker model.

**Figure 4:**
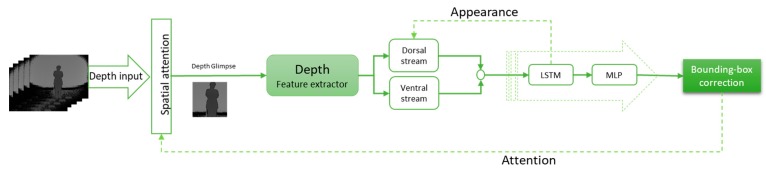
Depth tracker model. The glimpse is selected in spatial attention module, then using a CNN the appearance feature spatial feature is extracted and fed to LSTM from depth only.

The structure of the tracking module for RGB input is shown in Figure 2, RGBD models are presented in Figure 3, and the depth-only model is presented in Figure 4. The input is the RGB frame $\left(x_{i}\right),\;i\in \left\{1,\;\ldots ,\;f\right\}$ and/or depth frame $\left(d_{i}\right)\;,\;i\in \left\{1,\;\ldots ,\;f\right\}$, where f is the number of frames. The spatial attention extracts the glimpse ($g_{t})$ from these data as the part of the frame where the object of interest is probably located. The features of the object are extracted from the glimpse using a CNN. Two types of feature can be extracted from the glimpse ventral and dorsal stream. The ventral stream extracts appearance-based features $v_{t}$, while the dorsal stream aims to compute the foreground and background segmentation $s_{t}$. These features are then fed to a LSTM network and the Multi-Layer Perceptron (MLP). The output $o_{t}$ is a bounding box correction $\Delta \hat{b_{t}}$, and is then fed back to the spatial attention section ($a_{t+1}$) to compute the new glimpse and appearance $a_{t+1}$, in order to improve the object detection and foreground segmentation. Below, we explain each part in more detail.

*Spatial Attention:* The spatial attention mechanism applied here was similar to that of Kosiorek et al. [3]. Two matrices, $A_{t}^{x}\in R^{wxW}$ and $A_{t}^{y}\in R^{hxH}$, were created from a given input image, $x_{t}\in R^{wxW}$. These matrices consisted of a Gaussian in each row, whose positions and widths decided which part of the given image was to be extracted for attention glimpse.

The glimpse can then be defined as $g_{t}=\;A_{t}^{x}\;x_{t}\;{\left(A_{t}^{x}\right)}^{T}\;:g_{t}\;\in R^{hxw}.$ The attention was determined by the center of the gaussians (μ), their variance, σ and the stride between gaussians. The glimpse size was varied as per the requirements of the experiment.

*Appearance Attention*: This module converts the attention glimpse ($g_{t}$) i.e., $g_{t}=\;A_{t}^{x}\;x_{t}\;{\left(A_{t}^{x}\right)}^{T}\;:g_{t}\;\in R^{hxw}$ into a fixed-dimensional vector, $v_{t}$, which was defined with the help of appearance and spatial information about the tracked target (see Equation (1)).

(1) $$V1=\;R^{hxw}\;\to \;R^{h_{v}\;x\;w_{v}\;x\;c_{v}}$$

This processing splits into the ventral and dorsal stream. The ventral stream was implemented as a CNN, while the dorsal stream was implemented as a DFN (dynamic filter network) [38]. The former is responsible for handling visual features and outputs feature maps $v_{t}$, while the latter handles spatial relationships.

The output of both streams was then combined in the MLP module (Equation (2)).

(2) $$v_{t}=MLP\;\left(vec\;\left(v_{t}⊙s_{t}\right)\right)$$

where ⊙ is the Hadamard product.

*State estimation*: In the proposed method, LSTM was used for state estimation. It was used as working memory, hence protecting it from the sudden changes brought about by occlusions and appearance changes. 

(3) $$o_{t},h_{t}=LSTM\;\left(v_{t},\;h_{t-1}\right)$$

(4) $$\alpha_{t+1\;},\;\Delta a_{t+1},\;\hat{\Delta b_{t}}=MLP\;\left(o_{t},\;vec\left(s_{t}\right)\right)$$

(5) $$a_{t+1}=a_{t}+\tanh\left(c\right)\Delta a_{t+1}$$

(6) $$\hat{b_{t}}=a_{t}+\Delta \hat{b_{t}}$$

Equations (3)–(6) describe the state updates where c is a learnable parameter. In order to train our network, we have minimized the loss function, which in turn contains a tracking loss term, a set of terms for auxiliary tasks, and L2 regularization terms. Our loss function contains three main components, namely, tracking (LSTM), appearance attention, and spatial attention (CNN). 

The purpose of the tracking part was to increase the tracking success rate as well as the intersection over union (IoU). The negative log of IoU was utilized to train the LSTM network, similar to the approach introduced by Yu et al. [39].

The appearance attention was employed to improve the tracking by skipping pixels which do not belong to the target; hence, we define the glimpse mask as 1 where the attention box overlapped the bounding box and zero wherever else. The attention loss function was defined as the cross-entropy of the bounding box and attention box, similar to the approach adopted by Kosiorek, et al. [3]. 

The spatial attention determines the target in the scene; thus, the bounding box and the attention box should overlap to make sure that the target is located on the attention box. Meanwhile, the attention box should be as small as possible, hence we minimized the glimpse size while increasing its overlap with the bounding box. Based on intersection over union (IoU), we limited the number of hyper parameters by automatically learning loss weighting for the spatial attention and appearance attention. 

To add depth information to complement the RGB information, we have proposed three different structures. In the following sections, more details are provided for each of these methods.

### 3.1. RGD-Based Model 

To add depth information in this method, first we separated the RGB channels and transformed them into two channel spaces. Given the device’s three color channels (R, G, and B), the chromaticity coordinates r, g and b can be defied as: r =R−B, g =G−B, b =B−B=0. Thus, the last channel does not contain any information and can be easily skipped. Therefore, in our model, the r and g channels are utilized as the first two channels and the third channel is replaced by depth information. In this method, we did not change the original architecture of the RGB model; instead, the inputs were changed to convey more information without increasing the complexity of the model. Figure 3a demonstrates the structure of this approach.

### 3.2. RGBD Combined Model 

In this model, the depth map and RGB information were fed to an extended CNN using one layer of one-by-one convolution layer, whose output was then fed into the object detection model. The structure is shown in Figure 3b. In this method, the depth data were combined with RGB information using three one-by-one convolution layers to decrease the dimensionality from four channels (RGBD) to three. 

This model allows the feature extraction CNN to learn features that contain information from all four channels available. Also, since the number of learnable parameters is not significantly increased, a simpler architecture can be obtained. 

### 3.3. RGBD Parallel Model

In the last RGBD proposed model, we employed two distinct parallel CNN to extract features from the depth channel and RGB channels, respectively (Figure 3c). The extracted features from depth and RGB were later concatenated. Afterwards, using max pooling the most dominant features were selected. Since the nature of depth information is different to color information, having two different models to extract features for each of the channels seems to be the most accurate model. However, using this method increases the number of learnable parameters significantly.

### 3.4. Depth Model (DHART)

Until now, three methods have been discussed to utilize depth data along with RGB information. In this section, a tracking model was discussed to use only depth data for tracking. To extract features of depth information, four convolution layers were utilized, and similar to the previous method, the extracted features were fed into LSTM to track the target through time. The structure of this model is depicted in Figure 4.

## 4. Results and Discussions

In this section, we compare and discuss the four approaches proposed, along with the method proposed in [3]. The Princeton RGBD tracking dataset [4] was utilized to train and evaluate all models. The dataset divided the captured sequences into two sets of validation (for training) and evaluation (for testing) sets. In addition, we utilized our own captured videos by a single Kinect V2.0 sensor to evaluate the performance of each model on real-world scenarios. The first three layers of AlexNet [40] were modified (the first two max pooled layers were skipped) to extract features. For the DHART model, we utilized the same weight as AlexNet [40] for three middle layers. Considering the higher resolution of images taken from the Princeton dataset [4], the size of glimpse was set to 76×76 pixels, resulting in 19×19 features for both depth and RGB frames. For training, ten sequences from the Princeton RGBD tracking dataset [4] were selected, where each of the sequences had an average length of 150 frames and total frames of 1769 sequences in total. Training was performed on an Nvidia Geforce GTX 1060 GPU with 3GB of GRAM. The testing sets were selected from the dataset testing sequences, in which their main target was human, including 12 sequences with an average length of 120 frames each, and a total of 1500 frames. The models trained on the Princeton dataset were also tested on our own collected frames, which were grouped into five different sequences, with an average length of 70 on each sequence. Each collected sequence focused on one of the challenges of tracking, including color camouflage, depth camouflage, and depth-color camouflage. 

**Figure 5:**
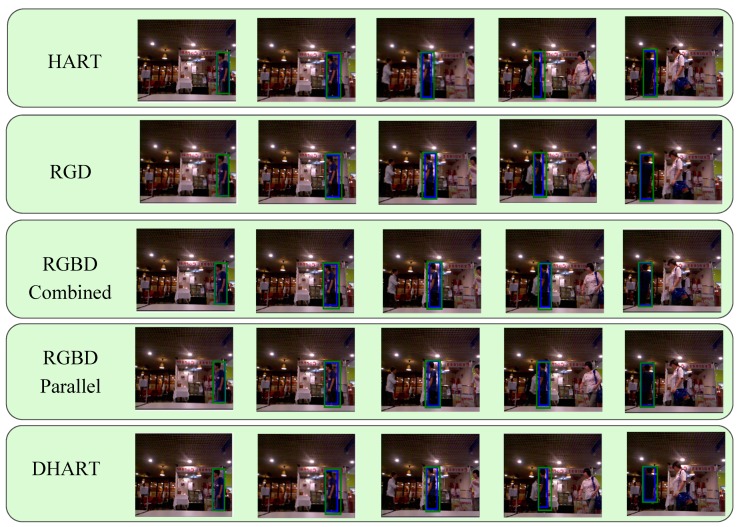
The object tracking model results with the Princeton dataset [4] (blue box—bounding box, green box—attention area): RGB, RGD, RGBD-combined, RGBD-parallel, and depth methods, from top to bottom, respectively. Each method successfully detected the target and tracked it. However, by using depth, the predicted bounding box was tighter.

**Figure 6:**
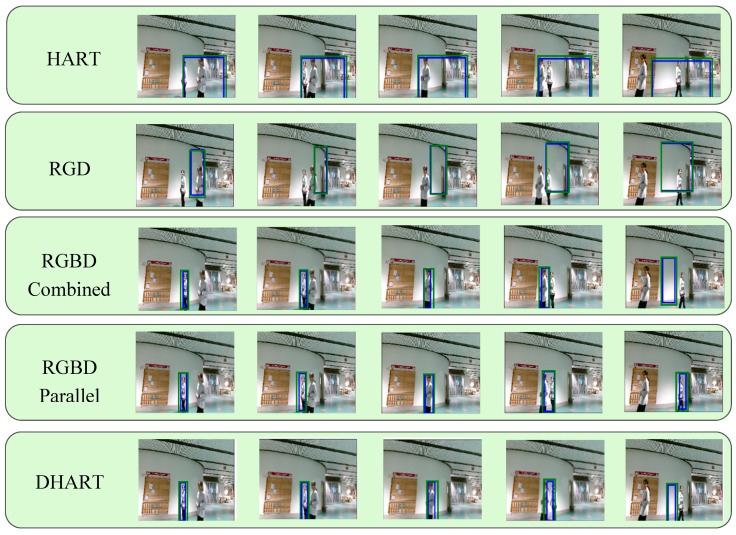
The object tracking model results (blue box—bounding box, green box—attention area): RGB, RGD, RGBD-combined, RGBD-parallel, and depth methods, respectively, from top to bottom. As can be seen, the color of the target, the background, and the occlusion are similar, and hence the HART algorithm was not successful tracking the target; however, the RGBD parallel model could track the target using extra depth features. Additionally, having only depth information performs better compared to having only RGB data.

An example tracking scenario and the predicted bounding box with each model is demonstrated in Figure 5; Figure 6, where Figure 5 is a selected sequence from the Princeton dataset and Figure 6 is a selected sequence from our collected data. It was observed that adding depth significantly improved the tracking results, especially when the foreground and background colors were similar. In Figure 5, the depth data were used to provide a tighter bounding box, giving a better accuracy. Figure 5 demonstrates a more challenging scenario with occlusion and color camouflage. When the tracking target was occluded by another person wearing a similar color (a color camouflaged scenario), the bounding box for the HART model becomes larger, as it assumes that the two humans are the same target. However, adding depth helps to distinguish the target better even than if the RGB profiles are similar. It was also shown that the RGBD Parallel structure is the most accurate tracking model. We also observed that depth information in itself shows a better result compared to RGB, RGBD combined, and RGD methods.

### 4.1. Results of the Princeton Dataset

The Princeton RGBD tracking dataset [4] provides a benchmark to compare different methods using four different metrics, that is, success rate, type I, type II, and type III errors. Type I and type III errors are defined as the rejection of a true null hypothesis (also known as a “false positive” finding). More specifically, type I error indicates the situation where the tracker estimates the bounding box far away from the target (Equation (9)) while the type III occurs when the tracker fails to indicate disappearance of the target in the scene (Equation (11)). On the other hand, type II error represents failing to reject a false null hypothesis (also known as a “false negative” finding) (Equation (10)).

More formally, we classify the error types as follows. The ratio of overlap between the predicted and ground truth bounding boxes $r_{i}$ is defined as follows [4]:

(7) $$r_{i}\;=\;\{\begin{array}{cc} IOU\left(ROI_{T_{i}},ROI_{G_{i}}\right)=\frac{area\left(ROI_{T_{i}}\cap^{\text{​}}ROI_{G_{i}}\right)}{area\left(ROI_{T_{i}}\cup^{\text{​}}ROI_{G_{i}}\right)} & \;ROI_{T_{i}}\neq \emptyset \;and\;ROI_{G_{i}}\neq \emptyset \;\\ 1 & \;ROI_{T_{i}}\;=ROI_{G_{i}}=\emptyset \\ -1 & otherwise \end{array}$$

where $ROI_{T_{i}}$ is the predicted bounding box in the frame number “*i*” (i.e., $x_{i}$ or $d_{i}$) in the sequence, $ROI_{G_{i}}$ is the ground truth bounding box, and $IOU\left(ROI_{T_{i}},ROI_{G_{i}}\right)$ is the intersection of the union. We set overlapping area $r_{t}$ to the minimum so that we could calculate the average success rate R for each tracker, as defined below [4]:

(8) $$R=\frac{1}{N}\overset{N}{\underset{i=1}{\sum^{\text{​}}}}u_{i}\;\mathrm{where}\;u_{i}\;=\;\{\begin{array}{cc} 1 & r_{i}>r_{t}\\ 0 & otherwise \end{array}$$

where $u_{i}$ indicates whether the bounding box predicted by the tracker for the frame number “*i*” is acceptable or not, *N* is the total number of frames, and $r_{t}$, as defined above, is the minimum overlap ratio to decide whether the prediction is correct or not. Finally, we can classify three types of error for the tracker as follows [4]:

(9) $$\mathrm{Type}\;I:\;ROI_{T_{i}}\neq \;\mathrm{null}\;\mathrm{and}\;ROI_{G_{i}}\neq \;\mathrm{null}\;\mathrm{and}\;r_{i}\;<\;r_{t}$$

(10) $$\mathrm{Type}\;\mathrm{II}:\;ROI_{T_{i}}\neq \;\mathrm{null}\;\mathrm{and}\;ROI_{G_{i}}\neq \mathrm{null}$$

(11) $$\mathrm{Type}\;\mathrm{III}:\;ROI_{T_{i}}\;=\;\mathrm{null}\;\mathrm{and}\;ROI_{G_{i}}\;=\;\mathrm{null}$$

The average success rate for our human tracking experiment, tested on the benchmark, is shown in Table 1. The success rate for tracking humans in the original HART model was only 29%. We were able to improve it to 46% using the RGD approach. In addition, the result of success rate for the RGBD combined approach and RGBD parallel approach was 39% and 48%, respectively.

Using the Princeton dataset benchmark, we were able to reduce type I error from 0.80 in the HART model to 0.73 in the RGBD-combined approach, and reduce it further to 0.62 in the RGBD parallel approach (Table 1).

We define precision of the tracking as follows:

(12) $$p_{i}=\frac{area(ROI_{T_{i}}\cap ROI_{G_{i}})}{area(ROI_{T_{i}})}$$

Hence, the average precision for each sequence, similar to Equation (8), is as follows:

(13) $$P=\frac{1}{N}\overset{N}{\underset{i=1}{\sum^{\text{​}}}}p_{i}$$

**Figure 7:**
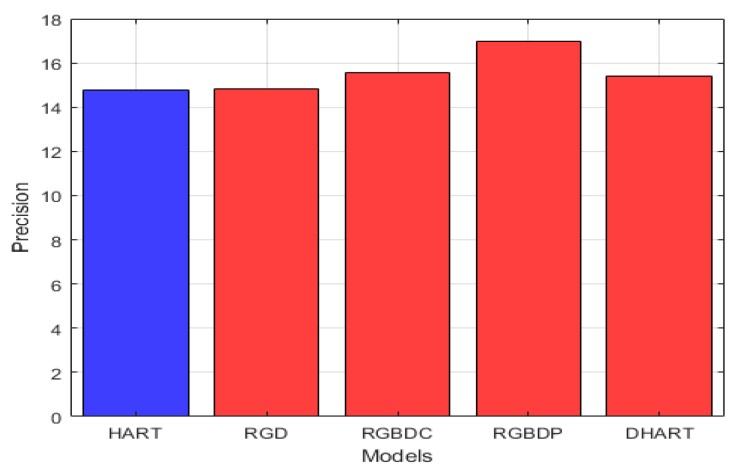
The comparison of mean average precision between HART and our four different approaches. The best approach is the RGBD-parallel model, which demonstrated an improvement of 15% over the original mean average precision of the HART model.

Figure 7 demonstrates the mean average precision of our four approaches in comparison to the original RGB tracker (HART). The improvement in precision for the RGBD-parallel, RGBD-combined, RGD, and DHART models over the HART model are 15.04%, 5.28%, 0.5%, and 4.4%, respectively. 

**Figure 8:**
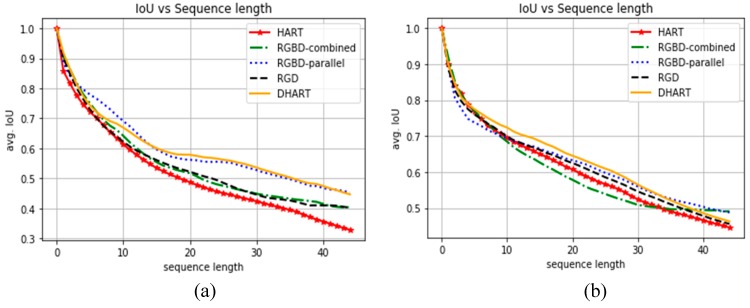
IoU curves on frames over 48 timesteps (higher is better). (**a**) Results for images captured by Kinect V2.0 in challenging environments containing color camouflage and occlusion. (**b**) Results for the Princeton Dataset. The results show that adding depth in all three methods improves the IoU, and using just depth also has a better performance on both real-word data and the Princeton dataset.

Figure 8b shows the average IoU curves on 48 timestamps in Princeton. To create these graphs for the Princeton dataset, the evaluation sets regarding human tracking were manually annotated. The results for the RGD, RGBD-parallel and DHART models are similar and have better performance than the HART model.

### 4.2. Kinect V2.0 Real-World Data 

We captured real-world data using a Kinect v2.0 sensor to evaluate the proposed approaches compared to the one proposed by Kosiorek et al. [3]. The employed metric is the IoU (Equation (7)). The results are shown in Figure 8a for our own evaluation sequences. As expected, by increasing the sequence length the IoU ratio dropped due to possible movements of the target and occlusions in the sequence. It is shown in Figure 8 that the IoU for RGBD-parallel and DHART yields the best results in both our evaluation dataset and the Princeton dataset. The IoU ratio stays over 0.5 even after 30 sequence frames for these methods, whereas it has a sharp decrease in the lower sequence lengths for the RGB based HART model. The RGBD-combined and RGD demonstrated a very similar performance in this plot, since their structures are very similar. The DHART model tends to have a better performance than the RGD and RGBD models. 

## 5. Conclusions and Future Work

In this paper, inspired by the RGB-based tracking model [3], we proposed three different RGBD-based tracking models, namely RGD, RGBD-combined, and RGBD-parallel, and one depth-only tracking model, namely, DHART. We showed that adding depth increases accuracy, especially in more challenging environments (i.e., in the presence of occlusion and color camouflage). To evaluate the proposed models, the Princeton tracking dataset was employed. The results of the benchmark showed that the success rate of the RGBD and DHART methods were 65% and 58% more than that of the RGB method, respectively. We also evaluated our models with real-world data, captured by Kinect v2.0 in more challenging environments. The results showed a significant improvement of the tracking accuracy when the depth is fed into the model. The results for the RGD and RGBD-combined models were similar. However, the RGBD-parallel and DHART model results demonstrated that these models are more efficient in object tracking compared to the other models. In future, the proposed approach can be extended to track multiple targets using the tracker for each of the objects in turn. 

## Figures and Tables

**Table 1:** Comparison between HART and our three different approaches.

Model	Type I Error	Type II Error	Type III Error	Success Rate
HART	0.80	0.073	0	0.29
RGD	0.60	0.069	0	0.46
RGBD Combined	0.73	0.069	0	0.39
RGBD Parallel	0.62	0.069	0	0.48
DHART	0.61	0.069	0	0.46
